# A wild fish image dataset for individual re-identification and phenotyping

**DOI:** 10.1038/s41597-026-07045-1

**Published:** 2026-03-18

**Authors:** T. K. Sørdalen, K. Malde, C. Sauvaitre, A. B. Skiftesvik, C. Beyan, T. Larsen, K. T. Halvorsen

**Affiliations:** 1 Institute of Marine Research, Bergen, Norway; 2 Centre for Coastal Research, Department of Natural Sciences, University of Agder, Kristiansand, Norway; 3 Department of Computer Science, University of Verona, Verona, Italy

## Abstract

Computer vision can transform wildlife monitoring by automating phenotyping and individual identification. Achieving this, however, depends on access to large, well-curated image datasets that capture natural variation across individuals and years. Here, we present Melops, a longitudinal dataset comprising 24 578 images of 9 861 individual corkwing wrasse, *Symphodus melops*, collected over seven years through a capture–mark–recapture survey. Each fish was PIT-tagged for re-identification and photographed from both sides against a standardized white background with a colour reference. Alongside the images, we provide metadata including body length, sex, and reproductive state. To support deep learning applications, the dataset includes both the original photographs and automatically cropped images focusing on the whole fish or specific body regions. Together, these resources provide a foundation for developing computer vision methods for individual re-identification, colour pattern analysis, sex classification and other visual phenotyping tasks. Beyond this species, Melops can serve as a model for similar datasets in other taxa. Because it contains thousands of individuals with repeated observations, it provides a rare opportunity to explore temporally aware re-identification and phenotypic change in wild fish.

## Background & Summary

Recent advances in artificial intelligence and computer vision have opened new possibilities for automating image analysis, species identification and phenotyping in studies of wild animals^1–3^. Another promising application is visual re-identification (re-ID) of individuals. Reliable re-ID could greatly benefit population monitoring, particularly for species that currently require the use of unique identifiers (tags) in mark-recapture studies^4,5^. Because tagging is costly, labour-intensive, and can affect animal behaviour or survival, there is growing interest in using natural “tags”, unique visual features such as stripes, spots, or other markings to identify individuals from photographs and video only.

Fish, however, present particular challenges for visual re-ID. They grow continuously throughout life, leading to substantial, often non-linear, changes in body shape and colouration over time. Many fish also exhibit seasonal or maturity-related variation in appearance, for example, secondary sexual traits such as bright colours or ornaments often develop during breeding season^6^. Individuals may also have transient marks such as wounds, fin damage, scars, or parasites that appear and disappear. Developing a robust re-ID model for fish, therefore, requires images that capture a wide range of natural variation and temporal changes in individual appearance. In practice, this calls for a large, longitudinal image dataset of known individuals sampled repeatedly over time coupled with new machine learning methods that can incorporate the temporal dimension and learn how the appearance of individual fish changes with age.

**Fig. 1:**
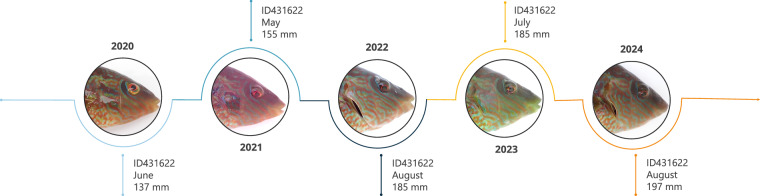
Example of repeated photographic encounters of the same individual nesting male corkwing wrasse (ID 431622) recaptured annually between 2020 and 2024. Despite differences in colouration and brightness across years, the timeline illustrates temporal stability in the male’s facial colour pattern, which forms the visual basis for individual re-identification in the Melops dataset.

**Fig. 2:**
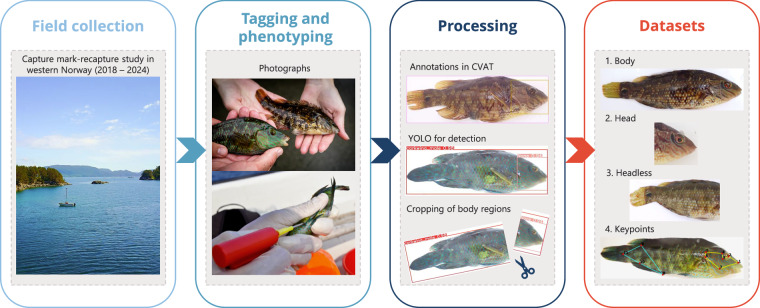
Overview of the Melops data workflow. Field collection involved capture, tagging, and phenotyping of individual corkwing wrasse, followed by standardized photography. Images were later processed through manual annotation in CVAT to define bounding boxes for the body and head regions, as well as 11 anatomical keypoints. These annotations were used to train YOLO models for automated detection and cropping of body regions, producing the final dataset.

Despite growing interest in developing such methods^1,7,8^, few openly available image datasets contain repeated photographs of the same individuals (high number of resightings; Figs. 1, 2, Table 1). This data scarcity has been recognized as a major bottleneck to progress in the development of automated re-ID methods^3,4^. Indeed, only a handful of deep learning-based re-ID studies on wild fish have been published so far^5,9,10^ likely reflecting this data deadlock. To address this gap, we present the *Melops* dataset, an extensive collection of annotated images designed for individual re-identification of the corkwing wrasse. Our primary motivation is to make this unique resource openly available to researchers worldwide so they can develop better re-ID approaches. Our second motivation is to encourage others to release similar datasets for other taxa to speed up the progress.

**Table 1:** Examples of shared wild fish re-identification datasets.

Dataset	Species	Images/Individuals	ID verification	Access
Whale Shark ID (LILA)^27^	Whale shark (*Rhincodon typus*)	~7 888 photos of 543 individuals	Visual	Public (open download)
USGS Brook Trout (EESC lab)^28^	Brook trout (*Salvelinus fontinalis)*	~435 individuals (each with one image annotation)	Tag	Public (open download)
MantaMatcher Database^29^	Reef manta (*Mobula alfredi*) and giant manta (*M. birostris*)	720 images of 265 individuals	Human (belly spots)	By request (not publicly shared)
NINA Brown Trout (NINA204)^30^	Brown trout (*Salmo trutta*)	Frames from 204 underwater videos; plus 288 images of 39 individuals	Tag	By request (research collaboration)
Undulate Skate Photo-ID^5^	Undulate skate (*Raja undulata*)	1 583 images of 108 individuals	Visual (dorsal pattern)	By request (not publicly shared)
Sharkbook/Wildbook for Sharks^31^	Multiple shark species	~94,920 sightings	Visual	By request (not publicly shared)

These datasets illustrate the range of species, sample sizes, accessibility, and methodological approaches for identification (Tag: independent from the animal’s visual traits vs Visual: human or machine-assisted identification on the animals visual traits). Together, they highlight the scarcity of openly available longitudinal image datasets for wild fish populations.

The Melops dataset comprises 24 578 images of 9 861 unique, wild-caught corkwing wrasse collected over seven years in Western Norway. This species is well-suited for visual identification: each individual has a facial “fingerprint”, a complex, high-contrast pattern on the head that appears temporally stable (Fig. 1). Because these patterns are not symmetrical, both the left and the right sides of each fish were photographed. Each fish was also tagged with a uniquely coded passive integrated transponder (PIT) upon capture, allowing for unambiguous re-identification if recaptured. To capture natural variation, individuals were sampled across a wide range of body sizes (and ages), from multiple locations, and during several seasons per year. The resulting dataset contains a high number of re-sightings, 8 527 images from 2 916 recapture events involving 1 882 individuals, providing the temporal depth needed to explore time-aware re-ID models (Fig. 1).

A preliminary study using a smaller subset of this dataset (2113 images from 513 individuals) evaluated individual re-identification using a contrastive metric-learning approach^9^. In that study, fish were first detected using a YOLOv5 object detector and cropped prior to re-identification. An embedding network based on a truncated Inception v3 architecture was trained using online hard triplet mining, and individual identity was inferred using nearest-neighbour classification in the learned embedding space. The dataset was randomly split into training and test sets, such that images of the same individual could occur in both sets across different capture events. Random splits do not explicitly enforce temporal independence and may therefore overestimate performance in longitudinal datasets where morphology changes through time^11^. The system achieved 35% one-shot (rank-1 accuracy) identification on a held-out test set, which increased to 53% when combining predictions from the left and right sides of the fish as an ensemble classifier. These results underscore the importance of accounting for sidedness, where separate models for each side can be trained and combined. The same study also reported higher accuracy when using head-cropped images compared with full-body images, highlighting the value of the facial region. Accordingly, the Melops dataset provides full-body, head and headless image crops, as well as 11 annotated key points enabling extraction of additional regions of interest.

Individual facial patterns are recognizable over time by human observers, as demonstrated by a validation of 135 individuals used for pairwise matching across time intervals ranging from 2 to 2 045 days (see technical validation section). To our knowledge, no deep learning–based animal re-identification studies have explicitly integrated or accounted for the temporal dimension. While some recent benchmarks employ time-aware data splits^8,11^ the underlying models remain time-agnostic. A few emerging multimodal frameworks, such as MetaWild^12^, have begun exploring metadata-based approaches that indirectly encode temporal or contextual cues. The results from Olsen *et al*.^9^ (authors, pers. comm.) reported that model performance declined with increasing time gaps between captures, likely reflecting the data bias toward shorter intervals between recaptures. Incorporating temporal dimensions directly, through multimodal architectures (e.g. Visual Language Models that combine temporal or contextual cues) or indirectly, by filtering implausible matches based on individually modelled growth trajectories, could improve long-term re-identification. The Melops dataset provides an ideal test bed for developing and evaluating such methods.

Beyond re-identification, the dataset also supports several other applications. First, it offers an opportunity to develop computer vision models for automated sex determination for this species. Corkwing wrasse have two male morphs, nesting males and sneaker males, that are fixed early in life. Nesting males are larger, more colourful, grow faster and provide paternal care, while sneaker males mimic females in appearance and behaviour^13,14^. Sneaker males can only be reliably distinguished from females during the spawning season when they release gametes. Because our sampling spanned both spawning and non-spawning periods, and because we recorded the sex of individuals confirmed by gamete release, the dataset includes a ground-truthed subset with verified sex labels. This subset also includes recaptures outside the spawning season, providing valuable data on seasonal variation in appearance for all sex. Such data can be used to train models that classify sex or identify sneaker males from subtle morphological cues, a focus of the study by Sørdalen *et al*. (in prep.).

Secondly, the dataset offers opportunities for colour pattern analysis. Wrasses are known for extraordinary intra-specific colour variability, which may reflect reproductive status, habitat background, diet, or social status. With thousands of images from repeated encounters, the Melops dataset allows for quantitative analysis of colour variation within and among individuals across time and space.

In summary, the Melops dataset fills a critical data gap for fish re-identification by providing a large, openly available set of individual-based, longitudinal images. We anticipate that this dataset will not only catalyse the development of more accurate and temporally robust re-ID models for fish, but will also support a broad range of ecological and evolutionary research on sexual dimorphism, alternative reproductive tactics, individual development and ageing, and colour variation in in wild corkwing wrasse populations. The framework is readily transferable to other species within the *Symphodus* genus, which has supported many fundamental studies on sexual selection and mating behaviour^15–17^.

## Methods

### Fish capture, tagging and photographing

Photographs and phenotypic data of corkwing wrasse were collected during scientific surveys conducted in Norway between 2018 and 2024 (Fig. 2). The majority of the images (92.8%) originate from standardized capture-mark-recapture surveys along the shorelines of three islands within a marine protected area (MPA) in Austevoll, western Norway. Wrasse were captured in shallow waters (1–8 m depth) using fyke nets or traps deployed overnight and hauled the following day. The capture position was recorded by a hand-held GPS (Garmin GPSMAP 78 s, Garmin International Ltd, Olathe, Kansas, USA). Fyke-net sampling occurred during three sampling periods each year with biological relevance: May (early nesting season), July (late nesting season), and August/September (after the peak growth season). Individuals larger than 100 mm total length were tagged with 11 mm passive integrated transponder (PIT) tags (RFID solutions), each with a unique six-digit numerical code readable with a handheld scanner (Biomark). Total length was measured to the nearest millimetre.

All fish were anesthetized using MS222 prior to tagging, and both sides of each individual were photographed in a white polystyrene box (25 × 25 × 25 cm inner dimensions). A few images from 2018 were taken using a custom-made box with black background. In 2019, photographs were obtained only during the September sampling period. Most photographs were taken with an *Olympus Tough TG5* or *TG6* camera using standard settings, however, mobile phones were occasionally used when the camera ran out of battery. In most images, a white balance card is visible to allow for colour standardization. Other wrasse species were occasionally photographed during these surveys but are excluded from the Melops dataset. All metadata was recorded manually in the field and digitized within 1–5 days after collection.

An additional 7.2% of the images were collected opportunistically during related field experiments and surveys between 2019 and 2023, using fyke nets, baited traps, or hand nets. For these samples, no trap identifiers (TrapID) are available and positions are approximate. Tagging and photographic procedures were as described above. The dataset also includes 15 untagged individuals from the AustNord location, which was only sampled once, and therefore excludes the possibility of encountering the same fish twice. For further details of capture, tagging and handling, see Halvorsen *et al*.^18^.

### Sexing

Nesting males can be reliably distinguished from females throughout life based on consistent morphological and colouration differences, particularly their facial patterns. In contrast, females and sneaker males are visually indistinguishable except during the spawning season (May-July) when gametes are released when gentle pressure is applied to the abdomen near the urogenital papillae. Outside the spawning period, all individuals with female-like phenotypes were classified as females with unvalidated sex (sex = 0) unless they had previously been sexed by gamete release during an earlier encounter.

### Ethics statement

All methods complied with Norwegian Food Safety Authority’s animal welfare laws, guidelines and policies as approved by the Norwegian Animal Research Authority (Application ID: 8715, 15307, 29473 and 30760).

### Image processing and cropping of regions of interest

#### Image annotation

To enable automated cropping and extraction of relevant body regions, 907 images were manually annotated using the Computer Vision Annotation Tool^19^. This annotated dataset primarily consisted of *S. melops*, also a small number of other wrasse species photographed during field sampling (Table 2); *Centrolabrus exoletus, Ctenolabrus rupestris, Labrus bergylta, Labrus mixtus*. For each *S. melops* image, sex was visually assigned as either (nesting) male or female; sneaker males were not separated from females for annotation purposes.

A subset of 505 *S. melops* images was further annotated with 11 anatomical keypoints and 4 keypoints marking the corners of the white balance reference card visible in most photographs. The 11 body keypoints defined two independent regions of interest (ROIs) for downstream applications such as colour analysis and re-ID:

- Head/cheek ROI: seven points; snout, eyes_snout, eyes_opercular, eyes_pectoral, top_opercular, medium_opercular, bottom_opercular.
- Abdomen-tail ROI: four points; anal_fin, caudal_top, caudal_bottom, black_point.

#### Automated detection of regions of interest and keypoints

A YOLOv8^20^ object detection model was trained to detect and predict bounding boxes for two regions, the fish body (snout to tail) and the fish head (snout to the rear edge of the opercula cover). The dataset was randomly split into 757 training and 150 validation images. The model was trained for 100 epochs on a single NVIDIA RTX 4090 GPU. The model was applied to the full image dataset to predict bounding boxes and infer species and sex.

We manually reviewed predicted labels on the new data, and any discrepancies between predicted species/sex and metadata were corrected in cases where obvious manual data punching errors were found. Only *S. melops* images were retained for the final dataset. The bounding box coordinates were then used to generate cropped images for head, body and headless body regions using the *magick* package in R^21^. YOLO failed to predict a bounding box for the *body* in one image, resulting in 24 577 cropped body and headless images compared with 24 578 original and head images. The relative position of the head in the frame was used to categorize images as left or right, verified by manual checks (a handful of images were captured upside down).

For anatomical keypoints, a YOLOv8-pose model was trained to predict the 11 keypoints. The dataset was split into 377 training and 126 validation images and trained for 100 epochs on a GPU. The trained model was applied to the full image dataset to predict keypoint coordinates. The model accuracy was evaluated by calculating Mean Normalized Error for each keypoint.

### Colour standardisation

Since the images are captured under natural variability in light conditions, we developed a python script for standardisation of brightness (L*) and the chromatic axes of green–red (a*) and blue–yellow (b*) using Python v3.12.7 within a Jupyter Notebook (Anaconda distribution). For each image, we calculated correction factors for these variables based on the deviation of the measured values of the white balance card from a reference standard (L* = 74, a* = 0, b* = 0)^22,23^. These may be used to correct the full image or any of the annotated body parts. The provided script demonstrates how correction can be applied to the ROIs defined by keypoints in the facial region. To this end, polygonal masks were generated with the polygon function from the skimage.draw package^24^, and pixel values within each ROI were extracted in the CIELAB colour space using OpenCV.

The Mean Colour Error (MCE) was then calculated using the CIEDE2000 formula, which quantifies the difference between two colours. Specifically, the MCE measured the distance between the observed white balance card values and the reference, both before and after correction. A reduction in MCE following correction indicates that the adjusted colour of the white balance card more closely matches the reference, confirming the success of the correction.

The CVAT-annotated datasets, the YOLO training and validation data, and Python and R scripts for bounding box, keypoint detection, and colour processing are available via the accompanying Zenodo repository (see Data Availability).

### Human evaluation experiment

#### Design and sampling of triplets

To assess human ability to re-identify individual corkwing wrasses from standardized images, we constructed a benchmark triplet–matching test (*FishFaces*). Each trial consisted of a query image (top) and two candidate images (bottom), one of which depicted the same individual (“correct” match) and the other a different individual (“wrong” match). Participants were asked to select which of the two candidates matched the query.

Triplets were generated from the full dataset of head-cropped images. Candidate pairs from the same individual were first identified using capture–recapture records. For each such pair, the earlier image was designated as the query, and the later image as the correct match. A wrong match was then sampled from a different individual of the same sex group and of similar body length (±5 mm), to reduce the possibility of trivial discrimination. Wrong matches were also preferentially drawn from the same season and year as the correct match, ensuring similar background conditions while varying the time elapsed between query and correct sightings.

To capture variation in temporal difficulty, pairs were stratified by the interval between query and correct images (dayseqdiff): Short: ≤ 150 days; Mid: 151–450 days; Long: ≥ 451 days. An equal number of triplets was sampled from each time bin within each sex group (males, females with validated sex, and sneaker males), resulting in a balanced design across sex and time interval. Within each triplet, the position (left or right) of the correct match was randomized.

Images flagged as blurry or smaller than 500 pixels in height were excluded from the dataset prior to Triplet selection. In addition, metadata including individual ID, capture date (as day-sequence), fish length, sex, year, and season were retained in an accompanying manifest file for downstream analysis. Each triplet was assigned a unique label (Q001, Q002, …). A total of 135 triplets were made for the benchmark test, though the sampling code can easily be modified for a different sample size.

#### Online form and participants

**Fig. 3:**
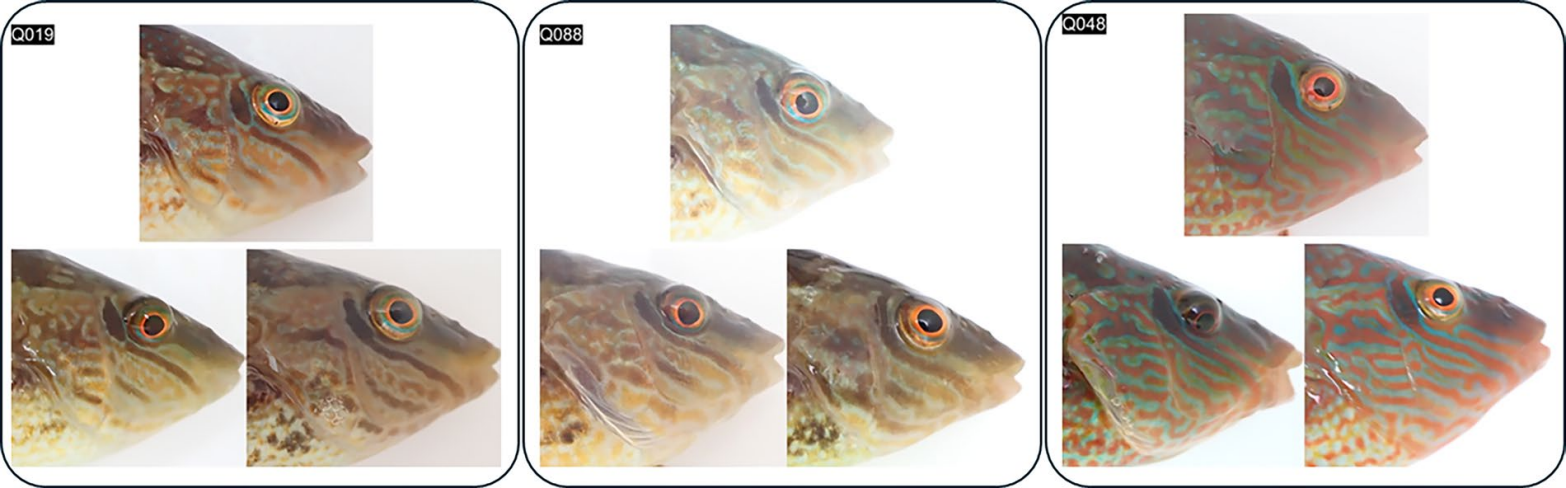
Three examples of triplets from the FishFaces human benchmark dataset. In each trial, the task is to select which of the two bottom images depicts the same individual as the top (query) image. The correct match (left) is shown for three cases: Q019 (female, 2 years and 309 days apart), Q088 (sneaker male, 2 years and 363 days apart), and Q048 (nesting male, 2 years and 63 days apart).

The benchmark was implemented as a Google Form, automatically generated from the manifest and the corresponding PNG triplet images using custom Apps Script. To prevent API timeouts, the script built the form in batches of 30 triplets (135 total). Each question presented one triplet on a separate page, the query image on top, and the two candidate images below, with participants selecting either “Left” or “Right” (Fig. 3).

The form was distributed among eight project collaborators with experience handling corkwing wrasse in the field or have been involved in other re-identification studies on this species. Results from this experiment are released together with the Melops image dataset as a human benchmark for evaluating machine learning models on the same re-identification challenge. Two of the eight human experts are not authors of this study but provided written informed consent to participate and to be named in the manuscript and associated submission files.

## Data Record

### Overview of data

**Fig. 4:**
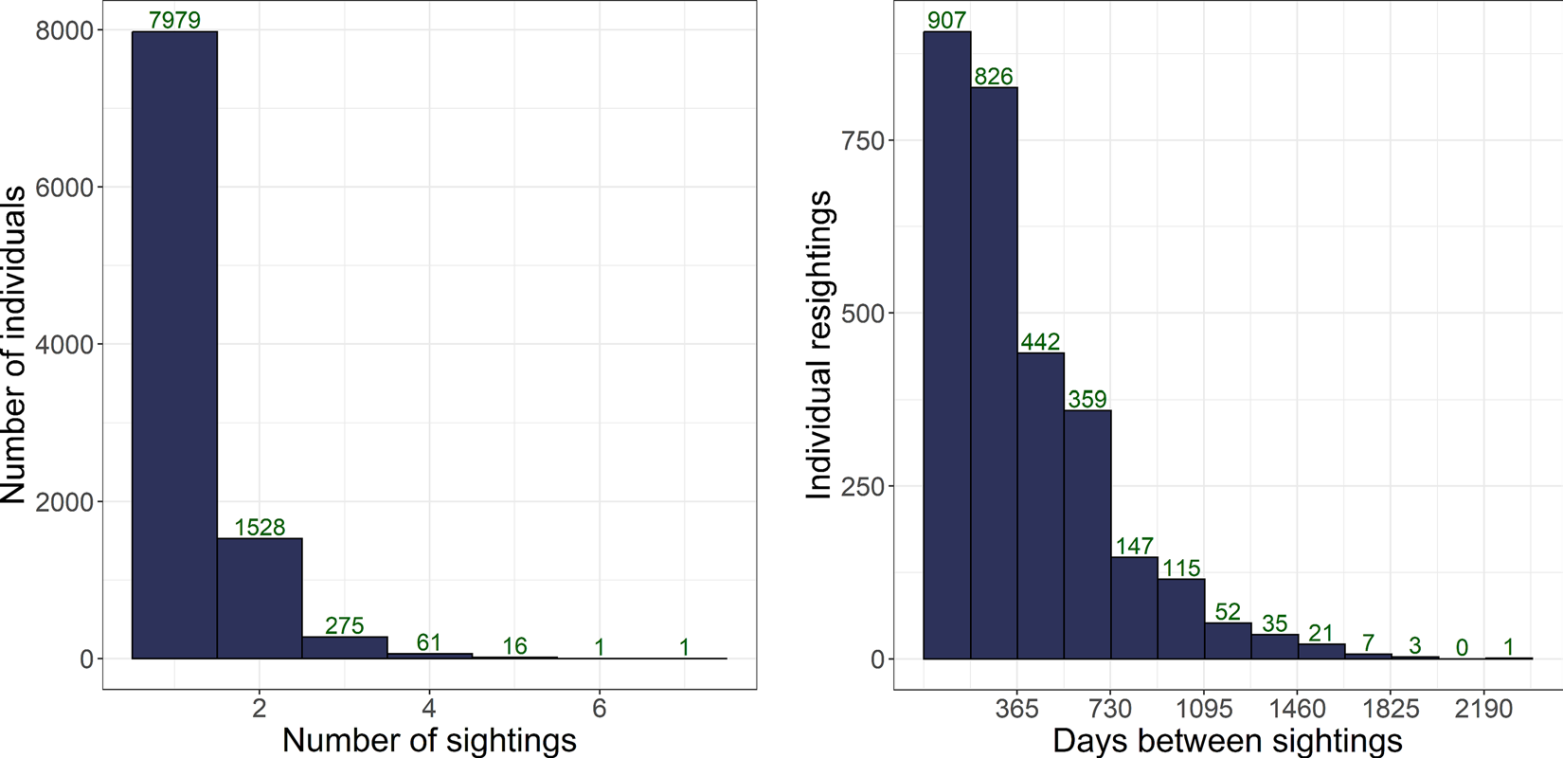
*Left:* The number of individuals and how many times they have been sighted in the Melops dataset. The majority of individuals have only been captured once (7 979), while 1 882 have been captured multiple times. *Right:* The temporal distribution of sight-resightings in the Melops dataset. Binwidth = 183 days.

**Fig. 5:**
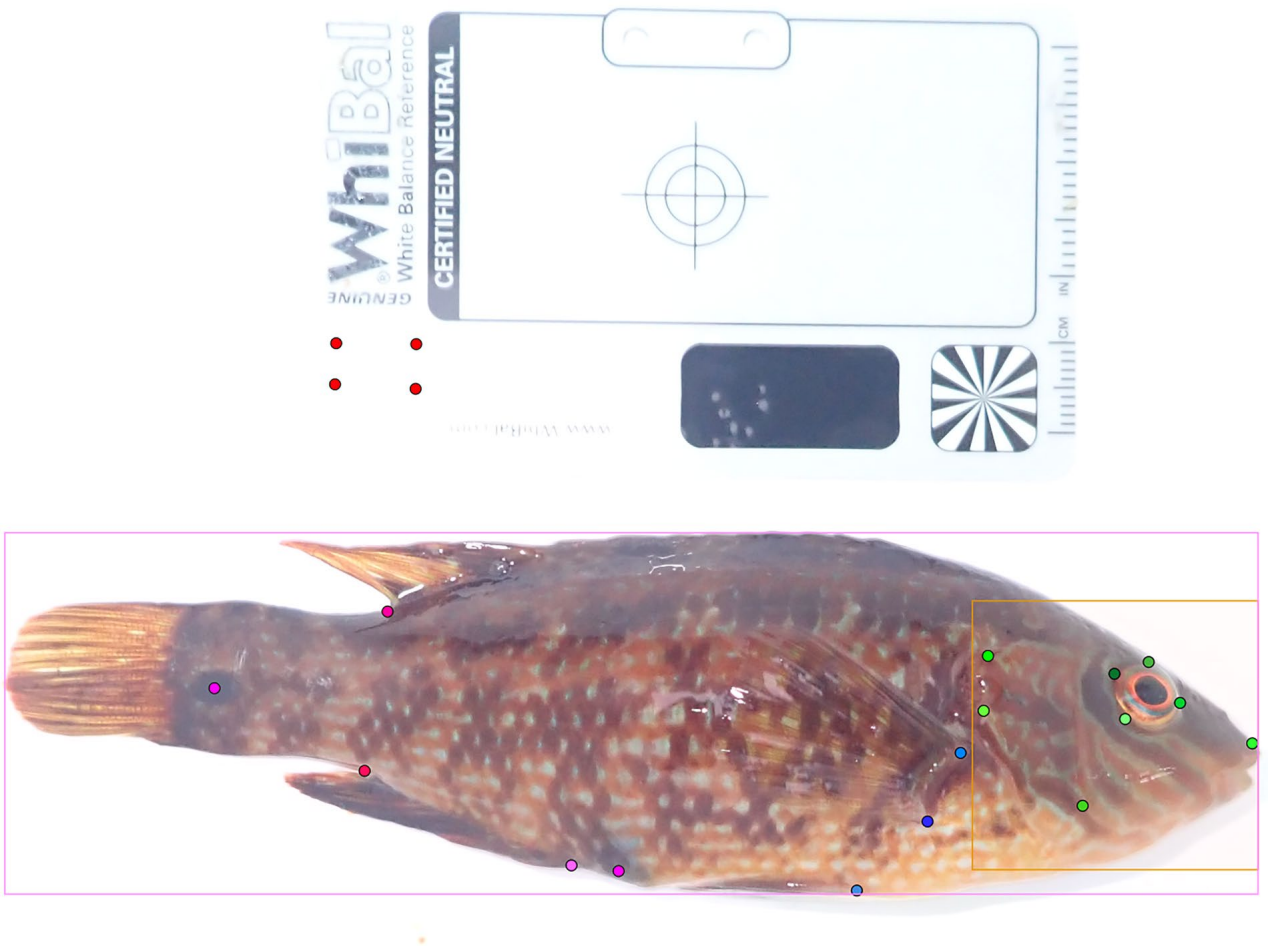
Example of a standardized photograph of a corkwing wrasse showing annotations created in CVAT, including full-body and head bounding boxes, 11 anatomical keypoints, and the colour reference card. Images were captured from the right side under background conditions.

The Melops dataset includes 24 578 images of 9 861 individual corkwing wrasse, of which 1 882 individuals were resighted at least once (2 916 resightings, 8 524 images; Fig. 4). The dataset and the code use to create it is available at zenodo^25^. The majority of resightings occurred within the same year (Fig. 5). The images are provided in four .jpg versions: full-sized originals and three cropped versions (body, head, headless). Most photographs also exist as raw.ORF files; however, due to file size constraints, these are not included in the online repository. Researchers wishing to access the raw files may contact the corresponding author.

Further, we provide relevant metadata, R and Python scripts for data handling and visualization and a manually annotated image dataset used to train machine learning models for automated detections of key points and regions of interest. Table 2 summarizes the main columns in the metadata file, including the individual ID with (pit-tag number), unique image identifier, and capture information (date, location, and sampling period). Ten individuals have *tagged* = = *0*, indicating that the tag was lost (evident from a tag wound) or that the fish was photographed but not tagged.

**Table 2:** Summary of columns in the Melops metadata file.

Column name	Description
filename_year	Unique image identifier (combines file name and year).
date	Capture date in “dd.mm.yyyy” format.
year	Capture year.
dayseq	Day sequence (running day number from 11.05.2018 = 1 to 30.08.2024 = 2305).
ID	Unique individual ID: PIT tag number (5–6 digits) or untagged followed by a number (1–15) for untagged fish.
tagged	Indicator if the fish is PIT-tagged (0/1).
suspected_tagloss	Indicator if the fish shows evidence of PIT-tag loss (0/1).
length	Total length in millimetres.
sex	f = female, m = male, s = sneaker male
validated_sex	Indicator if sex was validated (1 = validated, 0 = not validated).
sightings	Total number of sightings for a given individual (most sightings consist of two photos: left and right).
sightingnumb	Sequential number of each sighting for a given individual.
spawning	Spawning index based on gamete release when gentle abdominal pressure was applied during the spawning season (May–July). 0 = no gamete release; 1 = minor gamete release (few eggs or a small drop of sperm); 2 = substantial gamete release, indicating the individual was clearly ready to spawn.
cryptocotyle	*Cryptocotyle lingua* infection index (0–3), based on visual assessment of black spot disease caused by this digenean trematode parasite encysting in the skin: 0 = no visible cysts; 1,2.3; low, moderate or high density of visible cysts.
scaleloss	Index of scale loss, often a result of fights with conspecifics (0–3).
zombie	“Zombie” index (0–3), an unknown skin disease.
area	Sampling area – Sampling area (three islands within a marine protected area (MPA) in Austevoll: B = Bleikjo, L = Lambøya, S = Saltskjærholmen), plus two additional sites with fewer samples (AustNord, a separate MPA in Austevoll and Flodevigen, an MPA in Arendal, Southern Norway).
trapID	Identifier for trap or sampling device (e.g. 145_1).
lat	Latitude of sampling location.
lon	Longitude of sampling location.
side	Side of fish shown in image (left or right).
head_w/head_h	Width and height (pixels) of the head bounding box. Can be used to set quality thresholds.
blurry	Indicator if the image is blurry (1 = blurry, 0 = clear).

The table lists key variable names, descriptions, and data types associated with each image and individual. A complete column description, including all complementary metadata fields, is provided in the dataset’s readme file.

### Human benchmark dataset (FishFaces)

In addition to the core image dataset, we provide a benchmark dataset for assessing human performance in individual re-identification. This dataset (*FishFaces*) enables direct comparison between human and machine re-identification accuracy using standardized head images of *S. melops*. The benchmark archive is deposited alongside the primary dataset and includes the following components:

#### Triplet images

A set of 135 PNG images, each representing a triplet trial with query image (top) and two candidate images (bottom, left and right). One candidate shows the same individual as the query (the correct match), and the other shows a different individual of similar phenotype. The correct side (left or right) was randomized for each triplet.

#### Manifest file (manifest.csv)

A table containing metadata for each triplet, including triplet label (item_label; e.g. Q001), sex group, time bin (short, mid, long), and for each image (query, correct, wrong): individual ID, filename, fish length, capture year, season, and day-sequence. The file also specifies the position (left/right) of the correct match.

#### Answer key (answer_key.csv)

A simplified file mapping of each triplet label to the correct answer (“Left” or “Right”), derived directly from the manifest.

#### Response data (Human_benchmark_results.xlxs)

Responses from eight project participants (experts) on the FishFaces benchmark test. The participants; (wrasse experts, Msc or PhD: T; V). Six of the participants are authors of this paper and can be identified by their initials (TL; KH; CS; AB; TKS; AOT; KM), while Anil Osman Tur (AOT) and Vaneeda Allken (V) are project members at University of Verona and Institute of Marine Research, respectively funded by the parent project (CoastVision). Each row corresponds to a participants answer to a given triplet, with columns for respondent initials, item label, and response.

#### Human benchmark results (Human benchmark results.xlsx)

Columns include the triplet label (item_label; e.g. Q001) followed by the participants initials, with rows showing the participant’s responses and calculated scores (0–1).

Together, these files provide a reproducible benchmark for evaluating both human and machine performance in individual re-identification.

## Data Overview

Table 3 summarizes the number of individuals, sightings, and images by sex and morph, distinguishing between individuals with validated and unvalidated sex. Figure 4 shows the dataset composition and image content, including the number of individuals by sighting frequency, the temporal distribution of recaptures, while Fig. 5 illustrates a representative example of a standardised image with annotations in CVAT.

**Table 3:** Number of individuals, sightings and images for individuals in the dataset, separated on whether sex has been independently validated by stroking or is unvalidated individuals with female phenotypes.

	Validated sex			Unvalidated sex	Total
	Nesting males	Sneaker males	Females	(Females/sneaker males)	
n individuals	5297	318	807	3439	9861
n sightings	6549	442	1109	4097	12197
n images	13199	900	2264	8215	24578

Females and sneaker males cannot be visually distinguished outside the spawning season.

## Technical Validation

### YOLO detection and classification

**Fig. 6:**
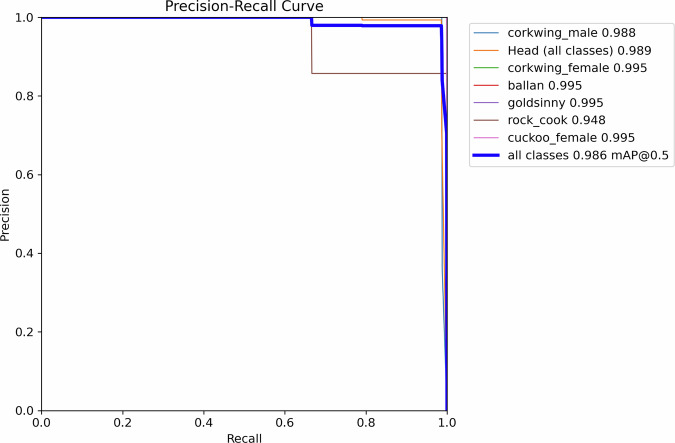
Precision–Recall curve for the YOLOv8x model trained to detect fish species and sex. The model achieved a mean average precision (mAP@0.5) of 0.986 on the validation dataset.

**Fig. 7:**
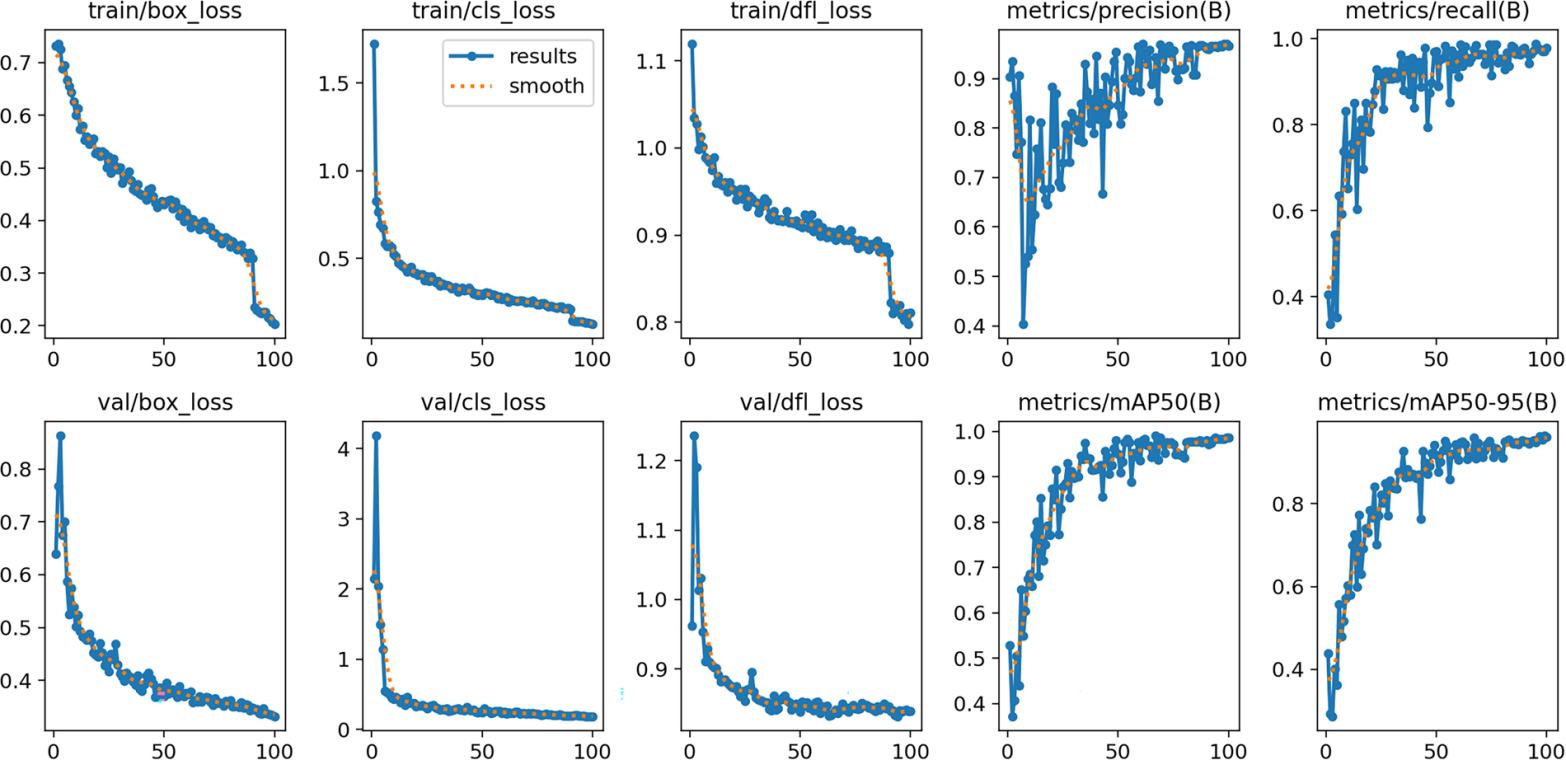
Training and validation losses across 100 epochs for the YOLOv8x model. The steady decline and stabilization of loss metrics indicate well-converged training.

The YOLOv8x model for body and head detection was trained to facilitate effective cropping of the full body, the head region, and the derived headless body. Each image contained a single individual, and annotations consisted of two bounding boxes: one enclosing the entire fish body and one enclosing the head region. Species and sex class labels were assigned to the body bounding boxes (species or species + sex for corkwing and cuckoo wrasse), while the head region was annotated as a separate generic class independent of species identity. Additional species were photographed and tagged alongside the focal species (corkwing wrasse), allowing the detector outputs to be used for filtering the image dataset by species or sex when required, and for verifying agreement between image metadata and model predictions. Because each image contained only one fish, only one body-class label was required per image. The class list comprised: corkwing_male, corkwing_female, corkwing_unknown, ballan, goldsinny, rock_cook, cuckoo_female, cuckoo_male, cuckoo_unknown, and head (internally labeled as “None” in the dataset configuration file).The model achieved high detection accuracy, with a mean average precision (mAP@0.5) of 0.986 and an F1 score of 0.97, indicating near-perfect precision and recall at the optimal confidence threshold (Fig. 6). Training and validation loss curves (Fig. 7) showed stable convergence without signs of overfitting. The trained detector provided accurate and robust bounding boxes for downstream automated cropping and phenotypic analyses.

### Keypoint detection

**Fig. 8:**
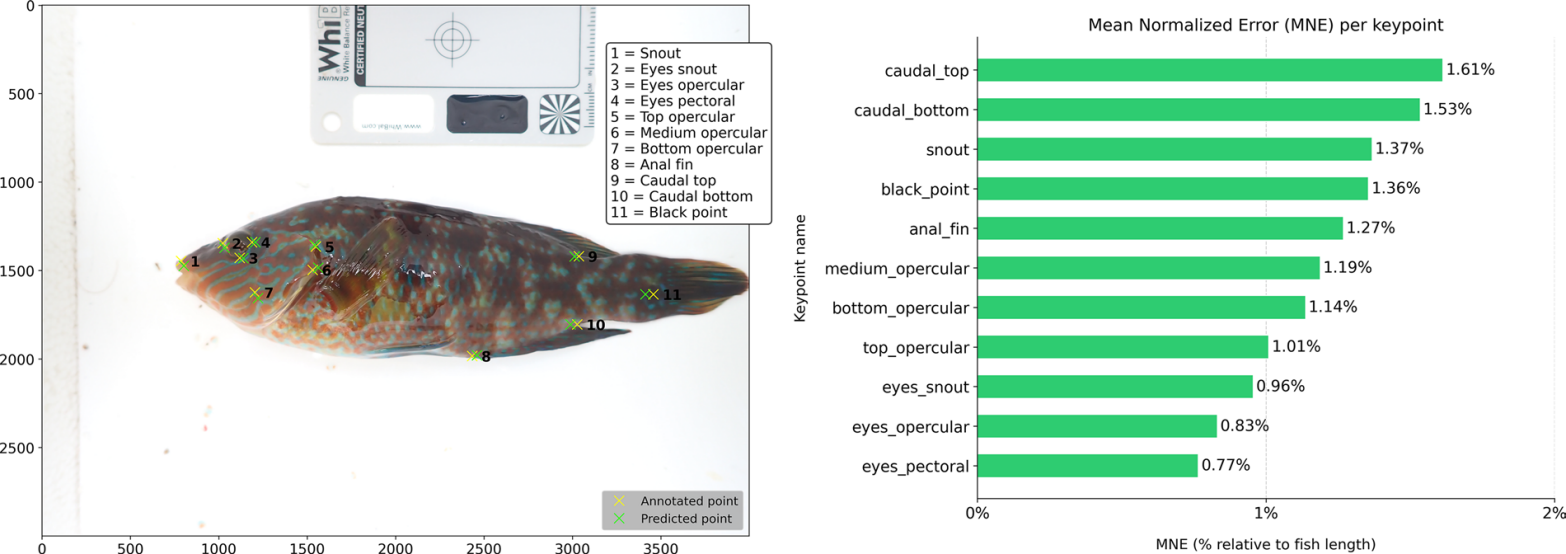
*Left:* Comparison between manually annotated (red) and model-predicted (blue) keypoints on a corkwing wrasse used to evaluate keypoint detection accuracy. *Right:* Mean Normalized Error (MNE) for each anatomical keypoint predicted by the YOLOv8 keypoint detection model, expressed as a percentage of fish length. Errors were calculated as the Euclidean distance between predicted and manually annotated keypoints, normalized by body length to allow comparison across individuals.

Model performance for anatomical keypoint detection was evaluated using the Mean Normalized Error (MNE), defined as the Euclidean distance between each predicted and manually annotated keypoint, normalized by the estimated relative body length of the fish. This normalization allows comparisons across individuals of different sizes. Relative body length was estimated from the maximum width of the bounding box enclosing each fish. An example comparison between manually annotated (red) and model-predicted (blue) keypoints is shown in Fig. 8, left. The low MNE values and visual alignment between predicted and annotated points demonstrate that the model accurately captured anatomical landmarks across varying poses and illumination conditions (Fig. 8, right).

### Image scaling consistency

**Fig. 9:**
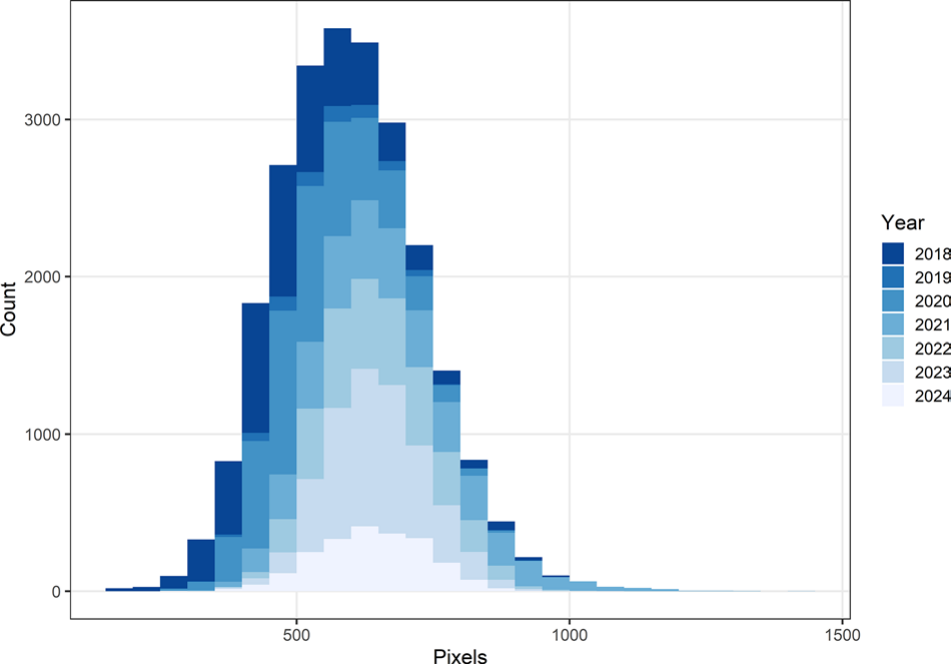
Variation in bounding box length across years. Differences in apparent size are attributed to variation in camera to subject distance during image capture.

To evaluate variation in image scale over time, the bounding box lengths produced by the YOLOv8x detector were compared across years (Fig. 9). Variability in box size reflects changes in the distance between the camera and fish during field photography rather than biological size differences, as each image was standardized to fill the frame. These observations provide transparency regarding imaging consistency over the study period.

### Human benchmark test

Performance across participants in the FishFaces benchmark test was consistently high (mean score: 132.25 correct out of 135, range: 129–135). Two participants achieved perfect scores, and four others made only one to four errors. All response data are provided in the file *Human_benchmark_results.xlsx*. Given this near-ceiling performance, no further stratified analysis by time interval or metadata was conducted. These results indicate that, although re-identification of *S. melops* requires fine-grained visual discrimination, human observers can achieve very high accuracy when distinguishing between two individuals. The FishFaces dataset thus represents a useful benchmark for machine learning models trained on the larger Melops_head dataset.

## Usage Notes

### Image quality

The *body* and *headless* datasets each contain one fewer image than the *head* dataset because the YOLO detector failed to identify the body in one case (P8316190_2020). We recommend excluding images that are flagged as “blurry” (59 images). The metadata includes image dimensions for all head crops, allowing researchers to decide whether to filter small image crops before model training or evaluation.

### Choice of crops

Preliminary analysis indicates that head crops outperform full body crops in open-set re-identification^9^. However, we encourage experimenting with all crop types, including combinations such as ensemble models that weight predictions from multiple body regions.

### Sidedness

Visual inspection of individual fish shows that the left and right sides display distinct but partially symmetrical facial patterns. For consistency, we recommend training and evaluating re-identification models using same-side image pairs (left–left or right–right), following the approach of Olsen *et al*.^9^. Predictions from side-specific models can be combined to form ensemble estimates if desired. While cross-side matching (left–right) is generally less relevant for standardized re-identification tasks based on controlled photographs, it may be useful for applications involving multiple camera views or video data.

### Colour

Images include a white balance reference card, allowing colour standardization using the grey area of the card. This could potentially improve re-identification and other classification tasks, and also enable analyses of spatio-temporal variation in colouration^26^.

### Spatial information

Spatial information (capture coordinates) can be used in filtering implausible re-identification matches. Corkwing wrasse have very limited movement^18^, making it unlikely for the same individual to be found more than 5 km apart or across deep channels separating the three islands (B, S and L).

### Setting up realistic experiments and case studies

When designing benchmark or case-study experiments, users may wish to reserve test subsets that reflect realistic temporal intervals, such as re-identifications between consecutive sampling periods (e.g., spring–summer or summer–autumn). Such setups can help evaluate model performance relative to known tagging data from closed-population surveys.

## Data Availability

The current version of the dataset is available at Zenodo (10.5281/zenodo.17404087). A summary of folders and files is provided in the readme.pdf. Additional data might be collected and uploaded in the future; in that case you can always find the most recent version of the dataset at Zenodo (10.5281/zenodo.17099924).

## Code availability

The code is available at 10.5281/zenodo.17404087. A summary of folders and files is provided in the readme.pdf.
